# Homebound Older Adults and Transportation Barriers to Social and Community Activities

**DOI:** 10.1093/geroni/igaf122.2483

**Published:** 2025-12-31

**Authors:** Kelly Vences, Angelina Gutierrez, Brian Fons, Namkee Choi

**Affiliations:** The University of Texas School of Social Work, Austin, Texas, United States; The University of Texas School of Social Work, Austin, Texas, United States; The University of Texas at Austin, Austin, Texas, United States; The University of Texas at Austin, Austin, Texas, United States

## Abstract

Although a homebound state in late life is often a result of cognitive and/or physical/functional health problems, transportation barriers may also be an important contributor. We used the 2023 National Health and Aging Trends Study (N = 7,543) to examine the associations of homebound states (defined as the past- month going-out frequency <1 a week) with driving status and self-reported transportation barriers to social/community activities. We used generalized linear models (GLM) to examine the associations. The results showed that homebound older adults comprised 5.2% of Medicare beneficiaries age 65 and older. Only 27% reported driving in the past month, and 54.2% and 32.1% reported health-related and transportation barriers to participating in social/community activities, respectively. GLM results were that homebound older adults were more likely to have stopped driving in the past year than their peers who went out 2-4 days a week (incident risk ratios [IRR]=2.28, 95% CI = 1.52-3.43) and those who went out 5+ days a week (IRR=7.20, 95% CI = 4.34-11.97). Homebound older adults were also more likely to report transportation barriers than those who went out 2-4 days a week (IRR=1.30, 95% CI = 1.06-1.58) and those who went out 5+ days a week (IRR=1.99, 95% CI = 1.50-2.65). Less than 10% of homebound older adults used taxi/ride-hailing services and public transportation. The study suggests that older adults’ homebound state significantly correlates with transportation barriers. Reliable transportation services appropriate to older adults’ capabilities are needed to facilitate their participation in social/community activities.

